# Defective Union in Fractures

**Published:** 1901-12-14

**Authors:** 


### Hospital Clinics and Medical Progress.

### DEFECTIVE UNION IN FRACTURES.

It is not easy to draw a definite line between delayed union and non-union of a fracture. To a certain extent this is a question of time, and it is a somewhat arbitrary proceeding to fix a period at which to say of a case of delayed union : Now at last this must be put down as a case of non-union. Still, if we are to draw distinction—and in view of treatment distinctions are quite necessary—this is all we can do. Mr. Tubby[1] says that if a simple fracture of the lower end of the tibia is not united in six weeks the case comes into the category of delay; but if, after three months' treatment, the callus is not ossified, it is a case of non-union. Even then, however, one has to remember that delay is almost normal in certain situations, so that it would not, for example, be correct to speak of delayed union at the end of six weeks after a fracture of the upper end of the tibia, for it is well known that healing in such a fracture is slow. Other points of distinction may be derived from the presence of callus or of pain on moving; for example, if some weeks after fracture a mass of callus be present and gentle passive movement causes pain and inflammatory signs, the case is probably one of retardation rather than failure of union. Absence of pain on passive movement suggests non-union, while presence of a large amount of callus generally indicates that union will ultimately take place. In regard to the treatment of delayed union it must be recognised that this condition not infrequently results from failure to secure the necessary immobility of the fragments, so that locally the most important measure is to immobilise the fragments effectively without undue pressure or interference with the circulation. In addition to this any causes of general malnutrition must be rectified. Common causes of this nature are syphilis, anæmia, and the debility arising from ague. To these must be added the debility produced by prolonged lying in bed, for in such cases union sometimes follows quickly upon the fixation of the fracture in plaster-of-Paris and the removal of the patient into the fresh air. In the treatment of non-union one must first eliminate the chance of the case being merely an instance of delay. One must be patient, improve the general health, immobilise the fracture, and wait. In some cases massage may be of use. But after patient trial of these means for several weeks, or even months, when the case becomes clearly one of non-union, the first thing is to dismiss from the mind as possible means of cure some of the modes of treatment which are still found in the text-books. "Such are the injection of irritating fluids, acupuncture, the introduction of setons and of foreign bodies and electrolysis. There are also doubtful methods spoken of, such as rubbing the ends of the fragments together and subcutaneous section of fibrous callus." All these methods are likely to lead to disappointment, and it is a pity that all mention of them is not expunged from the text-books. Aseptic surgery, however, puts certain resources at our disposal which are often attended with great success. These are, refreshment of the ends of the fragments, resection of the fragments and fastening them together, either by wire or screws, or by the application of a bone ferrule. In some cases transplantation of bone has been performed. Finally, we have to consider those cases in which while union occurs, it takes place in such a position as to interfere with the contour and the utility of the limb. In certain positions it may be sometimes impossible to avoid such mal-union. Such positions are the upper third of the femur and the middle of the humerus. But in most other cases the main causes of malunion are insufficient treatment at first and negligent treatment afterwards. Insufficient treatment at first too often means non-recognition of the fracture, but since the advent of the X-rays this ought no longer to occur. Negligent treatment means want of constant supervision afterwards, and the employment of unsuitable apparatus, such as ill-fitting splints, which fail to fix the fragments. The patient ought to be seen every two or three days in order to make sure that everything is going on well. In severe cases of mal-union one may have to consider the propriety of operation. Osteoclasis, that is "breaking the bone again," is not to be recommended. Its only advantage over osteotomy is the absence of a wound, but with proper precautions in regard to asepsis osteotomy is much to be preferred. Some cases occur, however, in which even the most carefully conducted operations fail to restore to bones their proper function.

[1] Brit. Med. Jour. Dec. 7.

